# Breastfeeding and the Longitudinal Changes of Body Mass Index in Childhood and Adulthood: A Systematic Review

**DOI:** 10.1016/j.advnut.2023.100152

**Published:** 2023-11-16

**Authors:** Miaobing Zheng, Ninoshka J. D’Souza, Linda Atkins, Saeed Ghobadi, Rachel Laws, Ewa A. Szymlek-Gay, Carley Grimes, Philip Baker, Qi-Qiang He, Karen J. Campbell

**Affiliations:** 1 School of Exercise and Nutrition Sciences, Institute for Physical Activity and Nutrition, Deakin University, Geelong, Australia; 2 School of Public Health, Wuhan University, Wuhan, Republic of China

**Keywords:** breastfeeding, obesity, BMI, longitudinal, trajectory

## Abstract

Mounting evidence supports the beneficial role of breastfeeding in lowering obesity risk, but the enduring impact of breastfeeding on longitudinal changes in body mass index (BMI) (i.e., BMI trajectories) remains unclear. This systematic review summarized evidence on how breastfeeding influenced the longitudinal trajectories of BMI later in life. Literature searches were conducted in Medline, Embase, Web of Science, and ScienceDirect to identify studies that assessed how breastfeeding (versus other feeding types or duration) was associated with longitudinal trajectories of BMI or BMI z-score. Three randomized controlled trials (RCTs) and 24 longitudinal cohort studies were included. Two-thirds (18/27) of studies were rated as acceptable or high quality. Most cohort studies (9/11) showed that children who continued to breastfeed at 3 to 12 mo of age had a lower BMI trajectory or lower odds of following a high BMI trajectory than those who were formula-fed or mixed-fed. The BMI differences between breastfeeding and other feeding groups were evident from age 7 mo and remained up to 8 y, and the magnitude of between-group BMI differences increased with age. For breastfeeding duration, 12 out of 15 cohort studies found that longer breastfeeding duration was associated with lower BMI trajectories up to age 18 y. Moreover, beneficial associations were observed for both exclusive and any breastfeeding with BMI trajectory. In contrast, mixed findings were reported from 3 RCTs that compared BMI trajectories from birth to ages 12 to 24 mo between breastfeeding promotion versus control or breastfeeding versus formula-feeding groups. The current review provides further longitudinal evidence from cohort studies that breastfeeding versus formula/mixed feeding or longer breastfeeding duration was associated with lower BMI trajectories. Such associations initiated in early childhood became more apparent with age and were sustained into early adulthood. The existing evidence substantiates the importance of breastfeeding promotion and continuation to support obesity prevention.

**Statement of significance**

To our knowledge, this is the first systematic review that critically evaluated the longitudinal evidence linking breastfeeding versus other feeding types or breastfeeding duration and trajectories of BMI over 3 or more time points later in childhood and adulthood.

## Introduction

Overweight and obesity are global health concerns. Substantial evidence suggests that obesity risk begins early in life and tracks into childhood and adulthood. In 2016, the global prevalence of overweight or obesity was 16% in children and adolescents and 39% in adults [1]. The prevalence is expected to rise if no actions are taken [1]. Understanding the early programming and determinants of obesity is vital for obesity prevention. A multitude of factors underlie the etiology of obesity. Nutrition in infancy and early childhood has significant impacts on a child’s growth and health later in life [2]. The rise of obesity reflects significant changes in human diets, including the displacement of breastfeeding with formula feeding [3]. Global estimates revealed that less than half of infants (44%) are exclusively breastfed [4]. Breastfeeding provides the best source of nourishment for infants. The pivotal role of breastfeeding in programming life-long health has been widely recognized [5]. World Health Organization (WHO) recommends that infants should be exclusively breastfed in the first 6 mo of life and continued for 2 y or longer alongside the introduction of safe and appropriate complementary foods. Moreover, WHO has recently set a global target of 70% for exclusive breastfeeding rate by 2030 to be achieved through comprehensive breastfeeding protection, promotion, and support policy measures [6].

The beneficial effect of breastfeeding in promoting optimal infant growth and protecting against later obesity risk has been endorsed by many existing systematic reviews and meta-analyses [4, [7], [8], [9], [10]]. However, the preponderance of the literature focused on the examination of the association between breastfeeding and obesity outcomes at only one subsequent follow-up. These studies provide no insights into the enduring impact of breastfeeding on longitudinal development of obesity. With the emerging availability of longitudinal data and the application of longitudinal statistical approaches, a growing number of studies have investigated the associations between breastfeeding and longitudinal changes in BMI, a universal measure of obesity, across the life course [11, 12]. These studies provide valuable insights into how breastfeeding influences the direction and the extent of change in BMI (e.g., trajectories) as well as the critical time points when the relationship emerged or is the strongest [11]. Longitudinal studies with measurements over >3 time points offer greater statistical power and precision of estimated effects and stronger evidence for inferring the temporal order (causal) of the relationship than prospective data with 2 time points [13, 14]. To date, no reviews have systematically reviewed and summarized the findings from these emerging longitudinal studies. Therefore, the current review aimed to systematically summarize studies assessing the role of breastfeeding in the longitudinal development of BMI over ≥3 time points in childhood and adulthood. The findings of this review will contribute further robust longitudinal evidence for breastfeeding guidelines and policies and will inform early obesity prevention interventions.

## Methods

### Eligibility criteria

The current review included studies that reported an association between breastfeeding and longitudinal changes in BMI or BMI z-score (e.g., trajectories). Eligible studies were required to assess longitudinal changes in BMI or BMI z-score as the outcome (≥3 time points). Eligible study design included randomized controlled trials (RCTs) and prospective cohort studies. RCTs could either involve breastfeeding promotion interventions or compare breastfeeding with other feeding types. Similarly, longitudinal cohort studies that assessed breastfeeding versus other feeding types or breastfeeding duration as the exposure of interest were included. Cross-sectional studies that collected data at 1 point in time were, thus, excluded. RCTs and prospective cohort studies evaluating the association between breastfeeding and changes in BMI or BMI z-score from baseline to a subsequent follow-up over 2 time points were excluded as findings of these studies have been summarized in previous systematic reviews and/or meta-analyses [[7], [8], [9], [10]]. Studies were limited to human studies published in English. Studies with children of very low birth weight (<1500 g), serious conditions, endocrine or metabolic disorders, or severe illness as the primary study population were excluded. The reporting of the current review followed the PRIMSA checklist, and the review protocol is registered at PROSPERO (CRD42021239367).

### Search strategy

Literature searches were conducted in Medline (PubMed), Embase, Web of Science, and ScienceDirect. Key search terms included: ([breastfeeding OR “breastfeeding” OR “breast-feeding” OR “breastfeeding duration” OR “infant feeding”] AND [“body mass index” OR “body mass index z-score” OR BMI OR “BMI z-score”] AND [“development” OR “trajectory” OR “trajectories” OR “longitudinal”]). Searches were conducted to gather literature from database inception to March 2023 with limits to original research articles. Literature searches were also conducted in Google Scholar using key terms to identify further eligible studies. The reference list of relevant studies was also searched to identify studies missing from database searches.

### Study selection and extraction

A two-step screening process was undertaken using Covidence with an initial title and abstract screening followed by full-text screening. Each study was screened by 2 reviewers (ND, MZ), and conflicts were resolved by discussion with a third reviewer (SG). Studies meeting all eligible criteria were retained for subsequent data extraction. The following information was extracted: study design, country, sample size, cohort name, gestational age, birth weight, type of breastfeeding variable, assessment of breastfeeding variable, how breastfeeding was analyzed, assessment of BMI, ages when repeated measurements of BMI were conducted, statistical analysis methods, potential confounders, main findings, and funding. Data extraction of each included study was undertaken by 2 independent reviewers (ND and MZ). Any differences in the extraction and interpretation of the data were resolved by discussion with a third reviewer (SG).

### Risk of bias assessment

Two researchers (ND and SG) conducted the quality assessment independently, with discrepancy resolved by discussion with a third reviewer (MZ). The Scottish Intercollegiate Guidelines Network 50 (SIGN 50) methodology checklists were used to evaluate the quality of the RCTs and prospective cohort studies. An additional 2 items on sample size justification and declaration of funding were added to the tool. The final checklist used for assessing quality of RCTs and prospective cohort studies contained 12 and 14 items, respectively. The final checklists aimed to evaluate the subject comparability, intervention/exposure, outcome, statistical analysis, and funding, which are 5 essential domains of good practice studies [15]. Studies that met most of the items (≥10 items for RCTs; ≥11 items for cohort studies) and unmet items that are unlikely to result in study flaws and influence the conclusion of the study was rated as high with no little/risk of bias. Studies were rated as acceptable if most items were met, but there were some flaws in the study design, and low if most items were not met with significant flaws in the study design.

## Results

### Study selection

**FIGURE 1:**
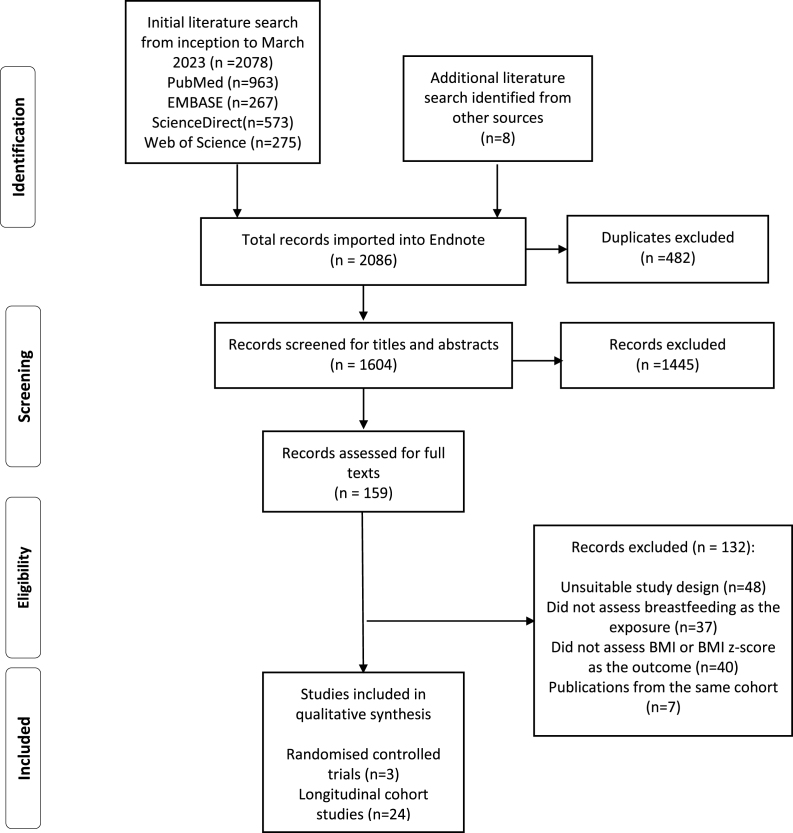
Flow chart for selection of studies examining the association between breastfeeding and longitudinal changes in body mass index in childhood and adulthood.

A total of 2086 citations were retrieved from 4 databases and other sources. After removal of 482 duplicates, 1604 were entered into Covidence for initial screening of titles and abstracts. Initial screening resulted in 159 papers being included in further full-text review, leaving 27 eligible studies for inclusion in the current review (Figure 1). Common reasons for exclusion included unsuitable study design, not assessing breastfeeding as the exposure and/or BMI or BMI z-score as the outcome, and duplicate publications from the same cohorts. For papers arising from the same cohort, the paper with the most recently published data was included in the current review. For example, both Chivers et al. and Oddy et al. [17] reported results from the same Australian cohort (Raine Study) [16, 17]. Oddy et al. [17], which report the most recent data with a longer duration of follow-up, were included in the current review, and Chivers et al. [16] were excluded.

### Study characteristics

Of the 27 included studies, 3 were RCTs [[18], [19], [20]], and 24 were longitudinal cohort studies (TABLE 1, TABLE 2). All 3 RCTs included healthy term European infants and had follow-up until ages 12, 18, and 24 mo, with sample sizes ranging from 141 to 17,064 [[18], [19], [20]]. Two RCTs compared the BMI trajectories of infants who were fed with different types of infant formula with a breastfeeding group as the reference group. One of these 2 RCTs involved high- versus low-protein infant formula [18], and the other RCT (COGNIS study) focused on standard versus enriched infant formula with bioactive nutrients [20]. The remaining RCT (PROBIT trial) evaluated the impact of breastfeeding promotion intervention on BMI trajectories of children in Belarus [19]. One RCT used both multilevel linear growth models and piecewise-linear regression models to assess BMI trajectories [18]; the other 2 RCTs used mixed effect models and ANCOVA, respectively [19, 20]. Two RCTs were adjusted for key potential confounders, including child sex, maternal education, or maternal BMI [18, 19]. In contrast, the remaining RCT did not adjust for any of these confounders [20].

**TABLE 1:** Randomized controlled trials assessing breastfeeding and BMI trajectories.^1^

Author, year	Sample	Intervention	Assessment of outcome	Ages at outcome assessment	Statistical methods	Adjusted confounders	Findings	Study quality
Koletzko [18], 2009	Multicenter European study *n =* 934, full term infants	B.F. vs. High protein formula vs. Low-protein formula	BMI z-score, health records and objectively measured	Birth, 3, 6, 12 and 24 mo	Multilevel linear growth models Piecewise-linear random effect models	Child sex, maternal education, smoking in pregnancy, country	High protein formula group had higher BMI than B.F. group at 6, 12, 24 mo, with greatest difference at 12 mo. No difference in BMI between B.F. and low-protein formula group. Compared with B.F. group, children fed with high formula had higher BMI z-score at 24 mo (0.20; 95% CI 0.05, 0.36).	High
Kramer, [19] 2018	Belarus *n =* 17046 PROBIT trial Healthy full term infants, B.W.≥2.5kg	B.F. promotion intervention vs. Usual care (Intention to treat) B.F. duration ≥12 vs. < 12 mo (observational)	BMI z-score, objectively measured	1,2,3,6,9 and 12 mo	Mixed effect models	Maternal education, infant sex, region, remoteness, maternal and paternal height, and BMI	Breastfeeding promotion group had higher BMI z-score at 1 mo (0.1, 95% CI 0.06, 0.13), 2 mo (0.1; 95%CI 0.07, 0.14), 3 mo (0.06; 95%CI 0.02, 0.09) than usual care group. No difference at birth, 6, 9, and 12 mo. B.F.≥12 mo vs. < 12mo had higher BMI z-score at birth, 1, 2, 3, 6 mo, but lower BMI z-score at 12 mo. No difference at 9 mo.	Acceptable
Sepúlveda-Valbuena [20], 2021	Spain *n =* 141, COGNIS study, full term infants	B.F. vs. Standard infant formula vs. bioactive nutrient-enriched infant formula	BMI and BMI z-score, objectively measured	2, 3, 4, 6, 12 and 18 mo	ANOVA, ANCOVA	Maternal age, height, intelligent quotient	No significant differences were found in BMI and BMI z-score among 3 groups.	Low

1 BMI: body mass index; B.W.: birth weight; full term ≥37 wk gestational age; B.F.: breastfeeding; Study quality assessed by Scottish Intercollegiate Guidelines Network 50 (SIGN 50) methodology checklists.

**TABLE 2:** Longitudinal cohort studies assessing breastfeeding and BMI trajectories.^1^

Author, year	Sample	Breastfeeding variable	Assessment of breastfeeding	Assessment of outcome	Ages at outcome assessment	Statistical methods	Adjusted confounders	Findings	Study quality
Buyken [29], 2008	Germany *n =* 434, DONALD, full term infants B.W.>2.5 kg	Any B.F. duration <4 mo vs. ≥4mo	Questionnaire at 3, 6,9,12 mo + 3-d WFR	BMI SDS, objectively measured	0.5, 1, 1.5, 2, 3, 4, 5, 6, 7 y	Linear mixed effects regression models	Maternal, age, education, overweight, smoking, gestational age, BMI SDS at birth, pregnancy weight gain, parity	B.F. ≥4 vs. < 4 mo showed lower BMI SDS trajectories in boys of overweight mothers. No association found in boys of normal weight mothers or girls.	Acceptable
Rzehak [30], 2009	Germany *n =* 7643 GINI-plus and LISA-plus, full term infants	Feeding groups Fully B.F. ≥4 mo (53%) vs. F.F./M.F.	Not reported	BMI, health records	0, 3, 6, 12, 14, 72 mo	Piecewise-linear random effect models	Maternal smoking, study center, parental education	BMI at birth and monthly BMI growth velocity in 5 growth periods 0–3, 3–6, 6–12, 12–24, 24–72 mo were lower in B.F. group vs. other feeding group, but the difference was small.	Low
Garden [21], 2012	Australia *n =* 370 CAPS study, GA>36 wk, BW>2.5 kg	Any B.F. duration 0 –3 mo vs. 3–6 mo vs. > 6 mo	Questionnaire every 3 mo in the first year	BMI, objectively measured	1, 3, 6, 9, 12, 18 mo, and every 6 mo thereafter until 5 y, and at 8 and 11.5 y	Growth mixture models and chi square test to assess association	None	Sex specific BMI trajectory groups derived. No relationship between B.F. duration and BMI trajectory groups.	Acceptable
Jwa [22], 2014	Japan boys *n =* 21425 girls *n =* 20147 LSB study, full term infants	Feeding groups E.B.F (22–23%) vs. M.F. vs. F.F. B.F. duration never vs. 1–2 mo vs. 3–5 mo vs. > 6 mo	Questionnaire at 6 mo	BMI, parent reported	0.5, 1.5, 2.5, 3.5, 4.5, 5.5, 7 and 8 y	Multilevel mixed effects model	Birth weight, sibling, maternal age, parental education level, household income, parental smoking	M.F. and E.B.F. infants had lower BMI trajectories than F.F. infants. B.F.>6 mo showed more BMI reduction from 1.5–7 and 8 y vs. never B.F.	Low
Oddy [17], 2014	Australia *n =* 2868 Raine Study, full term infants	Full B.F. duration <4 mo vs. ≥4 mo	Diaries of mothers for first 3 y	BMI or BMI z-score objectively measured	Birth, 1, 2, 3, 6, 8, 10, 14, 17 y	LGMM for BMI z-score trajectory from birth to 3 y; Linear mixed effect modeling for BMI trajectory from birth to 14 y, linear/logistic regression	LGMM: maternal prepregnancy BMI, education, smoking; LMM: maternal education	B.F. <4 mo increased the odds of following the early rapid growth class from birth to 3 y (OR: 2.05; 95% CI 1.43–2.94; p < 0.001); B.F.>4 mo showed lower BMI than ≤4 mo from 1 to 17 y.	High
Jensen [31], 2014	Denmark *n =* 311 SKOT cohort, full term infants	E.B.F. duration (mo) 0.5 mo vs. 4 mo Any B.F. duration (mo)	Questionnaire at 9 mo	BMI objectively measured	0.5, 9, 18 mo	Nonlinear mixed model (SITAR)	Child sex, birth weight, birth length	Longer E.B.F. (mo) was associated with an earlier BMI peak (-0.05; 95% CI, 0.07, -0.03) and lower prepeak velocity (-0.02; 95% CI, -0.02, -0.01) from ages 0.5 to 18 mo. No association found for any B.F. duration.	Low
Bell [36], 2017	USA *n =* 276, GA>35 wk	Feeding groups E.B.F. (70%) vs. exclusively F.F.	Monthly feeding diaries	BMI z-score, objectively measured	1, 4, 7 mo	Repeated measures ANOVA	Gestational age, child sex, ethnicity, maternal BMI, education study site	BMI z-score increased by 0.08 /mo (P < 0.001) in F.F. infants, whereas BMI z-score of B.F. infants remained constant (-0.005 /mo; P = 0.71).	Acceptable
Horodynski [40], 2017	USA *n =* 547, full term, BW2.5–4 kg	B.F. duration (B.F. termination age in mo)	Questionnaire at 2,6, 12 mo	BMI z-score objectively measured	Birth, 2, 6, and 12 mo	Growth curve models	None	Time since B.F. termination had negligible effect on BMI z-score growth velocity (0.001 95% CI, -0.027, 0.030).	Acceptable
Cheng [23], 2017	Hong Kong *n =* 7367 Children of 1997 birth cohort, no exclusion for G.A.	Feeding groups at 3mo E.B.F. (6%) vs. Mixed feeding vs. Formula feeding	Questionnaire at 3, 9, 18 mo	BMI z-score, objectively measured	0, 3, 9, 18, 24 and 36 mo annually from 6 to 16 y	Multivariable models using postestimation Wald tests Generalized estimating equations	Maternal smoking during pregnancy, household income, maternal education, age, maternal birthplace, parity, gestational age	No differences in BMI by feeding group for all age and sex groups.	High
Rzehak [41], 2017	Australia, Europe (pooled data) *n =* 3180 CHOP, HUMIS, PreventCD, no exclusion for GA	Full B.F. duration <3 mo vs. >3 mo	Questionnaire (details not specified)	BMI z-score, objectively measured	Every 6 mo from birth to 6 y	Latent growth mixture modeling	Gestational age, birth weight, maternal age at delivery, maternal prepregnancy BMI, maternal education, smoking during pregnancy	Infants with B.F. <3 mo had higher odds of being in the persistent rapid growth (OR: 3.80; 95% CI: 0.89, 16.24) and early rapid growth (OR: 1.65; 95% CI: 1.16, 2.35) rather than the normative-growth group.	Acceptable
Eny [12], 2018	Canada *n =* 5,905 TARGet Kids, full term, B.W. >1 kg	B.F. duration <6 mo vs. ≥6 mo	Questionnaire at multiple research visits	BMI z-score objectively measured	Birth and 1, 3, 18, 36, and 72 mo	Linear spline multilevel models	Child sex, birth weight, maternal BMI, maternal ethnicity, household income	Children who were breastfed <6 mo compared with ≥6 mo showed a higher growth rate between 1–3 (0.16, 0.11–0.21) and 3–18 mo (0.01, 0.005–0.002), and higher BMI z-score +0.24 (95% CI, 0.16, 0.33), +0.12 (95% CI, 0.02, 0.21), and +0.19 (95% CI, 0.007, 0.32) at 18, 36, and 72 mo, respectively.	Acceptable
Huang [24], 2018	China *n =* 1093 TMCHC, full term	Feeding groups E.B.F. (56%) vs. F.F. (low volume) vs. F.F. (high volume)	Questionnaire at 3 mo	BMI z-score (SDS), objectively measured	Birth, 3, 6, and 12 mo	Linear mixed effects model	Infant sex, infant birth weight, cesarean delivery, prepregnancy BMI and weight gain during pregnancy, and BMI z-score at birth	No difference in BMI z-score from birth to 3 mo. From 3–6 mo, BMI z-score of F.F. groups (0.12 and 0.15) was higher than B.F. (-0.01) (*P <* 0.05). BMI z-score at 12 mo of B.F. (0.5) was lower than F.F. groups (0.65 and 0.83) (*P <* 0.05).	Low
Liu [37], 2018	USA *n =* 2322 IFPS, GA>35 wk, BW>2.25 kg	Feeding groups - No B.F. - Initiated B.F., E.B.F.<4 mo - E.B.F. >4 mo, B.F. <12 mo - E.B.F. ≥4 mo and B.F. ≥ 12 mo	Multiple questionnaires during the first year	BMI z-score, parent reported	Birth, 3, 5, 7, and 12 mo	Latent class growth mixture modeling, multivariable logistic regression	Maternal age, ethnicity, marital status, education, income, smoking, prepregnancy BMI, gestational weight gain, child sex, birth weight, parity	E.B.F. for >4 mo and B.F.> 12mo was associated with lower odds of the infant is in the rising (OR: 0.17, 95% CI: 0.05–0.57) compared to the low-stable trajectory.	Acceptable
Iguacel [32], 2019	Spain *n =* 203 NEOBEFOOD Project, GA, not described	Feeding groups:M.F. (27%) (B.F./F.F.) vs. F.F. (73%)	Questionnaire at 9 mo	BMI z-score, objectively measured	6, 9 and 12 mo	Linear regression models	Child sex, parental education, BMI, child’s total food intake	BMI z-score of B.F. and F.F. at 6 and 12 mo not statistically different.	Low
Sherwood [33], 2019	UK *n =* 297/305 Isle of Wight Birth Cohort, U.K., no exclusion for GA	E.B.F, any B.F. (in wks)	Questionnaire at 1 or 2 y	BMI z-score, objectively measured	1, 2, 4,10, 18 y	GBTM, multinomial logistic regression	Child sex, birth weight, gestational age, maternal SES, maternal smoking, parity, maternal age	Any B.F. duration was associated with early transient overweight trajectory (log odds: -0.02, *P =* 0.002). No association with early persistent obesity or delayed overweight trajectories. E.B.F. duration showed borderline significance (-0.03, *P =* 0.05)	Low
Tian [25], 2019	China *n =* 927 GA 37–42 wk	E.B.F. duration at 6mo never vs. < 3 mo vs. 3–6 mo vs. >6 mo	Questionnaire at1,3,6 mo	BMI, objectively measured	1, 3, 6, 8, 12, 18, and 24 mo	Generalized estimation equation	Child sex, age, maternal and paternal age, education, mode of delivery, family income, birth weight, birth length	From 1 to 8 mo, E.B.F.>6 mo had higher BMI than other E.B.F. groups. From 12–24 mo, E.B.F.>6 mo had higher BMI than E.B.F. 3–6 mo (*P <* 0.05), but difference was small.	Acceptable
Zheng [26], 2020	Melbourne *n =* 483 InFANT, no exclusion for G.A.	Any B.F. duration < 6 mo vs. > 6 mo	Questionnaire at 3, 9,18 mo	BMI z-score, objectively measured	0, 3, 9, 18, 42 and 60 mo	Linear spline multilevel models	Child sex, birth weight, and gestational age; maternal country of birth, education, prepregnancy BMI and intervention group	Children who were breastfed for ≥ 6 vs. < 6 mo had lower BMI z-score at all ages from 3 to 60 mo. The adjusted mean differences in BMI z-score at 3, 9, 18, 42, and 60 mo were −0.34, −0.44, −0.13, −0.19, and −0.23, respectively.	Acceptable
Wu [35], 2020	UK *n =* 5266 British ALSPAC, no exclusion for GA	E.B.F. duration(mo) 0 vs. 5 mo 0 vs. 3 mo Any B.F. duration (mo)	Diary, interview at 6 and 15 mo	BMI health records, 10% measured	Birth, 10, 21, and 48 mo, and 7 y annually until 18 y	Mixed effects cubic spline model	Maternal BMI, education, smoking, gestational age (in mo)	E.B.F. ≥5 mo had lower BMI trajectories from 7 to 18 y than no E.B.F. Any B.F. duration showed weaker protective effects. Largest difference was seen at 18 y.	High
Flores-Barrantes [34], 2020	Spain *n =* 862 CALINAS study GA>36 wk	Feeding groups at 4 mo E.B.F. (38%) vs. F.F. vs. M.F.	Not reported	BMI z-score, objectively measured	Birth, 6 mo, and 1, 2, 3, 4, 5, and 6 y	Repeated measures ANOVA	Birth weight, gestational age, maternal education, maternal and paternal BMI, parental origin, and maternal smoking during pregnancy	F.F. infants had higher BMI z-score compared to B.F. and M.F. infants (P < 0.01).	High
Zheng [11], 2021	Australia *n =* 503 Healthy Beginnings Trial, GA ND	Feeding groups at 12mo B.F. (16%) vs. M.F. vs. F.F. B.F. duration < 6mo vs. ≥6 mo	Phone interview at 6 mo and face to face interview at 12 and 24 mo	BMI z-score, objectively measured, and medical records	Birth, 12, 24, 42 and 60 mo	Linear spline multilevel model (LSMM) GBTM	Intervention allocation, child sex, maternal smoking during pregnancy, marital status, education level, prepregnancy BMI	Both LSMM and GBTM showed B.F. compared to M.F./F.F., and duration (≥6 vs. <6 mo) had lower BMI z-score trajectory or lower odds of following the high BMI trajectory (P < 0.05).	Acceptable
Wang [38], 2020	United States *n =* 71892(retro study), no exclusion for G.A.	B.F. duration B.F.> 6 mo vs. ≤6 mo	Not reported	BMI, medical records	2–6 y	GBTM, multinomial logistic regression	Maternal age, education, race, child sex	B.F. ≤6 mo was associated with higher odds of following the high BMI trajectory group (OR 1.2 95%CI 1.2, 1.3) than B.F.>6mo.	Acceptable
Maskarinec [39], 2021	United States *n =* 269 M2M study, no exclusion on G.A.	B.F. and E.B.F duration 0–3 mo vs. 3–6 mo vs. 6–12 mo	Questionnaire at 12 mo	BMI z-score, medical records	Birth to 6y	Mixed effect quadratic model	Maternal age, race, ethnicity, education, marital status, parity, smoking during pregnancy, child sex, mode of delivery, birth maturity, age started daycare, birth weight	BMI z-score of children who were breastfed for 3–6 or 6–12 mo was lower by -0.70 (95% CI: -1.36, -0.04) or -0.50 (95% CI: -0.99, -0.01) than those who were breastfed for 0–3 mo. No evidence of an association found for E.B.F. duration.	Low
Chen[27], 2022	China *n =* 1649 full term LGA infants	Feeding groups at 1 y E.B.F. (40%) vs. F.F./M.F.	Questionnaire at 1 y	BMI z-score, objectively measured	Birth, 1, 2, 3, and 4 y	Mixed effect regression model with random intercept	Maternal prepregnancy BMI, maternal age, education, GDM, child sex	B.F. offspring had a significantly lower BMI z-score than F.F./M.F. (−0.06; 95% CI −0.12, −0.001, *P =* 0.047).	Acceptable
Longmore [28], 2022	Australia *n =* 258 PANDORA study, GA>34 wk	Feeding groups at 6mo Predominantly B.F. (70%) vs. non-B.F.	Phone/e-mail at 6–8 mo plus a subsample from medical records at 4–7 mo	BMI, medical records	Birth, 2, 8, and 14 mo	Mixed effect model with cubic regression splines	Not reported	Predominantly B.F. infants had lower BMI trajectories compared to nonB.F. infants, *P =* 0.006.	Low

1 BMI: body mass index; SDS: standardized deviation score; WFR: weighed food record; E.B.F.: Exclusive breastfeeding; B.F.: breastfeeding; F.F.: formula feeding; M.F.: mixed feeding; GA: gestational age; full term: ≥37 wk gestational age; GDM: gestational diabetes; LGA: large for gestational age; LSMM: Linear spline multilevel model; GBTM: group-based trajectory modeling; LGMM: latent growth mixture model LGMM; Study quality assessed by Scottish Intercollegiate Guidelines Network 50 (SIGN 50) methodology checklists.

Of 24 cohort studies, 23 included children from Asia Pacific nations (Australia, China; Japan, *n =* 10) [11, 17, [21], [22], [23], [24], [25], [26], [27], [28]], Europe (Denmark, Germany, Spain, United Kingdom; *n =* 7) [[29], [30], [31], [32], [33], [34], [35]], and North America (Canada, United States; *n =* 6) [12, [36], [37], [38], [39], [40]]. One study reported pooled results from 4 European and Australian cohorts [41]. This study included the Raine Study, which was reported by Oddy et al. [17]; therefore, results excluding the Raine study were extracted from supplementary results for inclusion in the current review. In summary, 9 studies assessed infant feeding types (breastfeeding versus other feeding types) as the only exposure [23, 24, 27, 28, 30, 32, 34, 36, 37], and 13 studies assessed breastfeeding duration (continuous or categorical) as the only exposure [12, 17, 21, 25, 29, 31, 33, 35, [38], [39], [40], [41]], and 2 studies assessed both exposures [11, 22]. Twelve studies evaluated exclusive breastfeeding versus other feeding types or exclusive breastfeeding duration [[22], [23], [24], [25], 27, 31, [33], [34], [35], [36], [37], 39], 6 of which clearly defined exclusive breastfeeding as per the WHO definition of breastmilk only without any additional drinks or foods including water [22, 24, 25, 27, 34, 35]. In contrast, 6 studies used different definitions [23, 31, 33, 36, 37, 39]. For instance, 1 study defined exclusive breastfeeding as breastmilk plus water and vitamins [31]. Exclusive breastfeeding was defined by Sherwood et al. [33] as breastfeeding until formula and solids were introduced [33]. In contrast, Bell et al. [36] defined exclusive breastfeeding as breastfeeding until the introduction of formula, regardless of solid food introduction. Three remaining studies provided no clear definition [[23], [37], [39]]. An additional 4 studies assessed predominant breastfeeding [17, 28, 30, 41]. Studies examined breastfeeding duration in both continuous and categorical forms. Five studies evaluated the association of continuous breastfeeding duration in weeks or months with BMI trajectories [31, 33, [40], [41], [42]]. Various breastfeeding duration cut-offs were used with most studies assessing a binary breastfeeding duration variable using cut-offs of 3 [41], 4 [17, 29], and 6 mo [11, 12, 26, 38]. Two studies examined exclusive breastfeeding duration of either 0.5 versus 4 mo [31] or 0 versus 5 mo [42]. Four studies grouped breastfeeding duration into 3 or 4 categories [21, 22, 25, 39]. For example, Jwa et al. [22], assessed breastfeeding duration of never, 1 to 2, 3 to 5 and > 6 mo [22]. Most studies assessed breastfeeding through questionnaires conducted at ≥ 1 time point, with the exception of 3 studies that did not report how or when breastfeeding was assessed [30, 34, 38].

Eight studies examined BMI as the outcome, whereas 16 studies examined the outcome of BMI z-scores. The age at the final outcome assessment ranged from 7 mo to 17 y with 5 studies in infancy (≤ 1 y), 5 studies in early childhood (2–5 y), 9 studies in midchildhood (6–9 y), and 5 studies in adolescence (10–18 y). Commonly used statistical methods to examine the association between breastfeeding and repeated measurements of BMI or BMI z-score were longitudinal trajectory modeling approaches: multilevel mixed effect models (quadratic, spline/piecewise, Superimposition by Translation and Rotation) and latent class trajectory analysis (growth mixture modeling, group-based trajectory modeling). These approaches account for the correlated data structure of the repeated measurements and use the exact age of measurements in deriving trajectories. Most studies used multilevel mixed effect regression to describe BMI or BMI z-score trajectories, whereby a model estimating the average BMI trajectory of the study sample was initially constructed. Breastfeeding variables were added into the model function as explanatory variables, enabling plotting of BMI trajectories by breastfeeding variables. Some studies conducted multilevel spline or piecewise model, which is an extension of the multilevel mixed effect model, where knots were defined and growth periods and specific growth velocities by breastfeeding were estimated. Studies that utilized latent class trajectory analysis identified heterogenous BMI trajectories within the sample, and the associations between breastfeeding and identified BMI trajectories were evaluated using logistic regression. Oddy et al. [17] and Zheng et al. [11] used both approaches. Other studies used linear mixed effect models, [27] generalized estimating equation (GEE), repeated ANOVA, and simple linear regression to make time point BMI comparisons [25, 32, 34, 36], and did not account for the correlated structure of longitudinal data. Commonly adjusted confounders were maternal education, maternal BMI, smoking during pregnancy, household income, child sex, childbirth weight, parity, and gestational age. Two cohort studies did not adjust for any covariates in their analysis [21, 40].

### Risk of bias

Overall study quality rating for each included study is shown in TABLE 1, TABLE 2. Detailed quality assessments of RCTs and longitudinal cohort studies for each SIGN-50 checklist item are presented in Supplemental Tables 1 and 2, respectively. Only 1 out of 3 RCTs included in this review was rated as high quality, meeting all checklist items [18]. The other 2 RCTs were rated as either low or acceptable quality because the criteria of randomization process, allocation concealment and/or intention-to-treat analyses were deemed unreliable [19, 20]. With respect to longitudinal cohort studies, most were acceptable (n =12) or high (*n =* 4) quality, [17, 23, 34, 35], with 8 low quality studies [22, 24, 28, [30], [31], [32], [33], 39]. Most studies addressed a clearly focused research question, involved similar populations, and defined clear study outcomes. Moreover, the assessment of outcome was blinded to exposure, and study outcomes were measured using valid and reliable methods and funding was declared. However, many studies did not provide adequate information pertaining to participation and dropout rates, or the comparison between participants and dropouts. Breastfeeding was self-reported by parents in all cohort studies and some studies that collected information across multiple time points received a higher rating than those assessing infant feeding at one time point. Studies of low quality also involved inadequate adjustment for confounding.

## Results synthesis

A summary of results from 27 studies by study design and type of breastfeeding exposure is presented in Table 3.

**TABLE 3:** Summary of results from studies that reported the associations of breastfeeding versus other feeding groups and breastfeeding duration with BMI or BMI z-score trajectories.^1^

Author, year	Country	Breastfeeding	BMI/BMIz trajectory	Age at final BMI/BMIz assessment	Significant protective associations	Study quality
***Randomized controlled trial (n = 3)***						
**Koletzko** [18]**, 2009**	European	B.F. vs. High protein F.F. vs. Low-protein F.F.	BMI/BMIz	2 y	Yes	High
**Kramer** [19]**, 2018**	Belarus	B.F. vs. usual care; B.F. duration	BMIz	12 mo	No	Acceptable
**Sepúlveda-Valbuena** [20]**, 2022**	Spain	B.F. vs. F.F.	BMI/BMIz	18 mo	No	Low
***Cohort studies (breastfeeding vs. other feeding groups) (n = 11)***						
**Rzehak** [30]**, 2009**	Germany	B.F.≥4mo vs. F.F./M.F.	BMI	6 y	Yes	Low
**Jwa** [22]**, 2014**	Japan	E.B.F. vs. M.F. vs. F.F. at 6mo	BMI	8 y	Yes	Low
**Bell** [36]**, 2017**	USA	E.B.F. vs. F.F. at 6mo	BMIz	7 mo	Yes	Acceptable
**Cheng** [23]**, 2017**	Hong Kong	E.B.F. vs. M.F. vs. F.F. at 3mo	BMIz	16 y	No	High
**Huang** [24]**, 2018**	China	B.F. vs. two F.F. groups at 3mo	BMIz	12 mo	Yes	Low
**Liu** [37]**, 2018**	USA	No B.F. vs. three B.F. groups	BMIz	12 mo	Yes	Acceptable
**Iguacel** [32]**, 2019**	Spain	Any B.F. vs. F.F. at 9mo	BMIz	12 mo	No	Low
**Flores-Barrantes** [34]**, 2020**	Spain	E.B.F. vs. F.F. vs. M.F. at 4mo	BMIz	6 y	Yes	High
**Zheng** [11]**, 2021**	Australia	Any B.F. vs. M.F. vs. F.F. at 12mo	BMIz	5 y	Yes	Acceptable
**Chen** [27]**, 2022**	China	E.B.F. vs. F.F./M.F.	BMIz	4 y	Yes	Acceptable
**Longmore** [28]**, 2022**	Australia	B.F. as the only milk at 6mo	BMI	14 mo	Yes	Acceptable
***Cohort studies (breastfeeding duration) (n =* 15*)***						
**Buyken** [29]**, 2008**	Germany	B.F. duration (< 4 vs. ≥4 mo)	BMIz	7 y	Yes	Acceptable
**Garden** [21]**, 2012**	Australia	B.F. duration (0–3, 3–6, >6 mo)	BMI	11.5 y	No	Acceptable
**Jwa** [22]**, 2014**	Japan	B.F. duration (never, 1–2, 3–5, >6)	BMI	8 y	Yes	Low
**Oddy** [17]**, 2014**	Australia	B.F. duration (< 4 vs. ≥4 mo)	BMI	17 y	Yes	High
**Jensen** [31]**, 2014**	Denmark	E.B.F. and B.F. duration (per mo; 0.5 vs. 4mo)	BMI	18 mo	Yes for E.B.F. No for B.F.	Low
**Horodynski** [40]**, 2017**	USA	B.F. duration (mo)	BMIz	12 mo	No	Acceptable
**Rzehak,** [41] **2017**	Australia/Europe	B.F. duration (< 3 vs. ≥3 mo)	BMIz	6 y	Yes	Acceptable
**Eny** [28]**, 2018**	Canada	B.F. duration (< 6 vs. ≥6 mo)	BMIz	6 y	Yes	Acceptable
**Sherwood** [33]**, 2019**	U.K.	Total B.F., E.B.F. duration (per week)	BMIz	18 y	Yes for B.F. No for E.B.F.	Low
**Tian** [25]**, 2019**	China	E.B.F. duration (never E.B.F., <3, 3–6, >6 mo)	BMI	24 mo	No	Acceptable
**Zheng** [26]**, 2020**	Australia	B.F. duration (<6 vs. ≥6 mo)	BMIz	5 y	Yes	Acceptable
**Wu** [35]**, 2020**	UK	E.B.F. and B.F. duration (per mo, 0 vs. 5mo)	BMIz	18 y	Yes for E.B.F. No for B.F.	High
**Zheng** [11]**, 2021**	Australia	B.F. duration (<6 vs. ≥6 mo)	BMIz	5 y	Yes	Acceptable
**Wang** [38]**, 2020**	USA	B.F. duration (≤6 vs. >6 mo)	BMI	6 y	Yes	Low
**Maskarinec** [39]**, 2021**	USA	E.B.F. and B.F. duration (0–3, 3–6, 6–12 mo)	BMIz	6 y	Yes for B.F. No for E.B.F.	Low

1 BF: breastfeeding; E.B.F.: exclusive breastfeeding; F.F.: formula feeding; M.F.: mixed feeding; USA: United States of America; UK: United Kingdom. Significant protective associations with *P <* 0.05; Jwa [22], 2014 and Zheng, 2021 assessed both breastfeeding versus other feeding groups and breastfeeding duration.

### Randomized controlled trials

#### Breastfeeding versus other feeding types and BMI or BMI z-score trajectories

Two RCTs compared the BMI trajectories of formula-fed infants in reference to breastfed infants in the first 2 y of life and showed inconsistent findings. Koletzko et al., [18] rated as high study quality, found infants fed with high protein infant formula, but not low-protein infant formula, had statistically significantly higher BMI z-score at ages 6, 12 and 24 mo than the breastfed group. In contrast, Sepúlveda-Valbuena et al. [20] rated as low study quality, observed no significant differences in BMI among 2 infant formula groups and the breastfeeding group from ages 2 to 18 mo. The third RCT of acceptable quality conducted by Kramer et al. [18] revealed that the breastfeeding promotion group had higher BMI z-score at ages 1, 2, 3 mo than the usual care group (*P* < 0.05), but no between-group differences were found at birth, 6, 9 and 12 mo [19].

#### Breastfeeding duration and BMI or BMI z-score trajectories

In addition, Kramer et al., [19] also compared the BMI z-score trajectories by breastfeeding duration. Children with breastfeeding duration ≥ 12 versus < 12 mo had higher BMI z-scores at birth, 1, 2, 3, 6 mo, but lower BMI z-score at 12 mo. No difference was found at age 9 mo.

### Cohort studies

#### Breastfeeding versus other feeding types and BMI or BMI z-score trajectories

There were 11 studies that assessed the association of breastfeeding versus other feeding types with BMI (*n =* 3) or BMI z-score trajectories (*n =* 8). Nine studies of varying study quality (1 high, 4 acceptable, and 4 low quality) found exclusive or predominant breastfeeding groups showed lower BMI or BMI z-score trajectories up to ages 7 [36], 12 [24, 37], and 14 mo [28], and 4 [27], 5 [11], 6 [30, 34], and 8 y [22] when compared with other feeding groups. These 9 studies assessed exclusive or predominant breastfeeding at ages 3 [24], 4 [30, 34, 37], 6 [22, 28, 36] and 12 mo [11]. Of which, 6 compared a breastfeeding group with formula and mixed-feeding groups combined [27, 28, 30] or as separate groups [11, 22, 34]. Two studies compared the breastfeeding group with 1 formula feeding group [36] or with 2 formula-feeding groups (low versus high volume) [24]. Liu et al. [37] examined a composite infant feeding variable combining breastfeeding exclusivity and duration. Specifically, these 9 studies revealed that breastfeeding groups showed slower BMI growth rates [30, 36], lower BMI trajectories [11, 22, 24, 27, 28, 36], and lower odds of following a high BMI group [11, 37].

Two of 11 studies with high or low quality found no evidence of an association between breastfeeding versus other feeding groups and BMI z-score trajectory [23, 32]. Cheng et al. [23] compared BMI z-score trajectory from birth to age 16 y by infant feeding group at age 3 mo in Chinese children using mixed effect regression models and reported the exclusive breastfeeding group, mixed-feeding group, and formula-feeding group showed similar BMI z-score trajectories. Iguacel et al. [32] compared BMI z-score from birth to age 12 mo between infants who were breastfed (including both breastfed and formula-fed) versus formula-fed at age 9 mo and found no differences in BMI z-scores between 2 groups using linear regression in Spanish children.

#### Breastfeeding duration and BMI or BMI z-score trajectories

Of 15 studies reporting the associations between breastfeeding duration and BMI or BMI z-score trajectories, 12 studies (2 high, 6 acceptable, and 4 low study quality) found longer breastfeeding duration was associated with lower BMI trajectories up to ages 1.5 [31], 6 [38], 8 [22], and 17 y [17] or BMI z-score trajectories up to 5 [11, 26], 6 [12, 39, 41], 7 [29] and 18 y [33, 35]. Mixed findings were reported from the 4 studies that assessed both exclusive and any breastfeeding duration [31, 33, 35, 39]. Jensen et al. [31] demonstrated that longer duration of exclusive breastfeeding (in months), but not any breastfeeding duration, was associated with an earlier BMI peak and lower prepeak BMI growth velocity from ages 0.5 to 18 mo in Danish children. Similarly, Wu et al. [35] revealed that a longer exclusive breastfeeding duration (≥ 5 versus 0 mo) showed a stronger association with lower BMI trajectory from birth to age 18 y than any breastfeeding duration, and the association was stronger when children were older with the largest difference observed at age 18 y in a British cohort. In contrast, Sherwood et al. [33] found any breastfeeding, but not exclusive breastfeeding duration (in weeks), was associated with higher odds of following the high BMI z-score trajectory from ages 1 to 18 y. Likewise, Maskarinec et al. [39] found significant associations between a longer ‘any breastfeeding’ duration (3-6 and 6-12 versus 0-3 mo) and a lower BMI z-score trajectory from birth to age 6 y, but not for exclusive breastfeeding duration. Eight studies assessed any breastfeeding duration alone; 3 studies found children who were breastfed for ≥6 versus <6 mo showed lower BMI trajectories from birth to age 5 or 6 y in Canadian and Australian children [11, 12, 26]. Wang et al. [38] revealed that American children who were breastfed for ≤6 mo had higher odds of following the high and mid BMI groups than the low BMI trajectory group from ages 2 to 6 y. Similar results were found in a study of Australian children where breastfeeding duration ≥6 versus < 6 mo was linked with lower odds of following the “High BMI z-score” trajectory [11]. In 2 other studies, significant association between breastfeeding and BMI trajectory was also revealed when breastfeeding duration was categorized using 4 mo [17, 29] and 3 mo [41]. Buyken et al. [29] found significant associations of breastfeeding duration ≥4 mo and lower BMI z-score trajectories in German children, but only in boys of overweight mothers. In a large cohort of Australian children, Oddy et al. [17], showed breastfeeding duration <4 mo increased the odds of following the early rapid growth trajectory group from birth to 3 y; children who were breastfed for >4 versus ≤4 months showed lower BMI trajectories from ages 1 to 14 y, and higher BMI at 17 y. Rzehak et al [41]., found short breastfeeding duration < 3 mo was associated with being in persistent rapid growth and early rapid growth rather than the normative-growth BMI z-score trajectory group from birth to 6 y. Jwa et al. [22], compared the BMI trajectory by various breastfeeding duration categories (never, 1–2, 3–5, > 6 mo) and showed that children who were breastfed for 1 to 2, 3 to 5, and > 6 mo versus never breastfed had lower BMI at ages 5.5, 7, and 8 y, and no differences in BMI were found at ages 1.5, 2.5, 3.5, and 4.5 y.

Of 15 studies, 3 studies of acceptable quality did not report protective effects of longer breastfeeding duration on BMI or BMI z-score trajectories [21, 25, 40]. In a cohort of Chinese children, Tian et al. [25] documented that exclusive breastfeeding duration > 6 mo had a higher BMI than other groups (never, ≤3, 3–6 mo) from ages 1 to 8 mo. Moreover, from ages 12 to 24 mo, exclusive breastfeeding duration > 6 mo had a higher BMI than exclusive breastfeeding for 3 to 6 mo (*P* < 0.05), but the difference was small. The other 2 studies found no evidence of an association between any breastfeeding duration and BMI trajectories in Australian [21] and U.S. children [40], respectively. Garden et al. [21] demonstrated that breastfeeding duration (0–3, 36, and > 6 mo) was not associated with BMI trajectory groups from ages 1 to 11.5 y. Horodynski et al. [40] found that breastfeeding duration had little impact on BMI z-score growth velocity from birth to age 12 mo. Of note, all 3 studies adjusted for minimal or no covariates, and median duration of breastfeeding was 2 mo [40] or not reported [21, 25].

## Discussion

To our knowledge, this is the first review that systematically summarized the longitudinal evidence linking breastfeeding (versus another feeding type; duration) and subsequent BMI trajectories over ≥ 3 time points in childhood and adulthood. Extending the findings of previous reviews [[7], [8], [9], [10]]. The current review provides new insights into the critical time points when a beneficial association of breastfeeding with BMI emerged, how the association changed over time, and whether such association remained consistent or was sustained into later life. Results from cohort studies showed that children who were exclusively or predominantly breastfed between 3-6 mo of age had a lower BMI trajectory than those who were formula- or mixed-fed. The between-group differences in BMI increased with age and were evident from age 7 mo up to 8 y. With respect to breastfeeding duration, most cohort studies reported consistent associations between a longer duration of breastfeeding and lower BMI trajectories up to age 18 y. Moreover, protective associations were found for both exclusive and any breastfeeding duration, and the association was evident for breastfeeding duration of 1 to 2 mo. However, the enduring impact of breastfeeding on BMI trajectories cannot be drawn from RCTs due to the short duration of follow-up and mixed findings.

The beneficial role of breastfeeding versus formula feeding in obesity prevention is well-recognized. Previous meta-analysis of data from 25 studies spanning 12 countries showed obesity risk was 22% lower among breastfed children compared to never-breastfed children[43]. Additionally, exclusively breastfed children had 20% reduced obesity risk than exclusively formula-fed children[44, 45]. Our review contributes further longitudinal evidence to support the beneficial association of breastfeeding versus formula-feeding or mixed-feeding on BMI trajectories.

It is widely accepted that longer duration of breastfeeding protects against obesity development in children. Whether a dose-response relationship exists between breastfeeding duration and obesity risk has received widespread attention. Many cohort studies included in our review investigated if BMI trajectories differed by categorical forms of breastfeeding duration. In contrast to previous meta-analyses of cohort studies that revealed a linear dose-response relationship between longer breastfeeding duration and lower risk of childhood obesity [10, 43, 46], our review reported inconsistent findings. Studies assessing breastfeeding duration using 3, 4, or 6 mo as a cut-off found children with breastfeeding duration > versus ≤ 3 to 6 mo showed a lower BMI trajectory [11, 12, 17, 26, 29, 41], indicating a potential threshold effect. In contrast, a few other studies examined the associations between breastfeeding duration (with 3 or 4 duration categories) and BMI trajectory and demonstrated discrepant findings, with 2 reporting a potential dose-response relationship [22, 39] and the other 2 reporting no significant associations [21, 25]. Hence, the consensus regarding whether a dose-response association exists between breastfeeding duration and the BMI trajectory cannot be drawn from our review.

Our review provides additional evidence addressing the controversy as to whether the beneficial association of breastfeeding with BMI differs by exclusive or any breastfeeding. Most cohort studies included in our review revealed significant associations between any breastfeeding duration and BMI trajectory. There were a few cohort studies that compared the association of any or exclusive breastfeeding with the BMI trajectory, but these showed conflicting findings. This is likely due to variations in how any or exclusive breastfeeding was defined or captured (whether solid foods or other fluids were considered). The totality of evidence from our review suggests that both exclusive and any breastfeeding showed beneficial associations with the trajectory of BMI.

A small proportion of cohort studies (5/24) found no associations of breastfeeding versus other feeding types or breastfeeding duration with BMI trajectories [21, 23, 25, 32, 40]. Of note, these studies had lower breastfeeding rates (e.g., lower proportion of sample in the breastfeeding group), lower median duration of breastfeeding, shorter duration of follow-up, did not use longitudinal trajectory modeling approaches, or adjusted for minimal or no potential covariates when compared to studies that found significant associations. For instance, one study reporting null association for exclusive breastfeeding versus other feeding groups had an exclusive breastfeeding rate of 6% [23] versus a range of 22%–70% in studies reporting significant associations [22, 24, 27, 28, 30, 36]. The other study that found no BMI differences between breastfeeding and formula-feeding children included formula-feeding infants in the breastfeeding group [32]. Further, these 2 studies did not use longitudinal trajectory modeling, but GEE or linear regression to compare repeated measures of BMI among the feeding groups. Compared to traditional GEE or linear regression, longitudinal trajectory modeling makes better use of repeated data that characterizes change over time by estimating mean or individual trajectories of a variable (e.g., BMI), captures nonlinear changes, accounts for between-person variations, accommodates missing data, and has greater statistical power [47]. For 3 studies that found no evidence of association between breastfeeding duration and BMI trajectories, 2 studies had follow-up in early childhood at age 12 and 24 mo, respectively [25, 40]. Informed by our findings that the beneficial impact of breastfeeding appeared to strengthen with age, it is conceivable that the enduring impact of breastfeeding may have not emerged by 2 y of age. The remaining study did not adjust for any covariates in their analyses, and residual confounding may in part, have contributed to null findings [21].

Several putative mechanisms have been proposed to explain how breastfeeding protects against the development of obesity [48]. The most common hypotheses point toward the potential mediating effect of rapid weight gain during infancy and breastmilk composition. Previous research has shown that breastfed infants tend to have slower weight gain compared to formula-fed infants during infancy [49]. Compelling evidence has shown rapid weight gain during infancy is highly predictive of later overweight or obesity [50, 51]. A recent pooled analysis of 7 cohorts demonstrated that the association between breastfeeding duration and BMI z-scores in childhood was fully mediated by infant rapid weight gain [52]. The differential composition of breastmilk compared to infant formula, such as lower protein content and the presence of bioactive factors, may also lend itself to protect against obesity. A systematic review concluded that high protein intake during infancy is associated with high BMI z-scores and elevated obesity risk later in life [53]. Furthermore, bioactive factors such as appetite-regulating hormones, e.g., ghrelin, adiponectin, and leptin, that are present in breastmilk may contribute to appetite regulation and prevent excess energy intake, in turn reducing risk of obesity in the long term [54]. For instance, research has shown grehlin, a catalyst for growth hormone secretion, shared an inverse relationship with weight gain among breastfed children, but this was not observed among formula-fed children [48]. Additionally, research has revealed that children who were breastfed > 4 mo had greater dietary variety or quality compared to those with shorter breastfeeding durations or who never breastfed, which in turn has been shown to lower obesity risk [[10], [48]]. Longer duration of breastfeeding accentuates these potential beneficial pathways, thereby posing stronger protection against obesity. In addition, children with a longer duration of breastfeeding may be less likely to be introduced to solid foods before 4 mo of age, which is a potential risk factor for obesity [55]. Future investigation of the underlying mechanisms supporting the beneficial role of breastfeeding in development of obesity is warranted.

Our review has several strengths and limitations. The inclusion of longitudinal studies with BMI measurements over 3 or more time points is a major strength of this review. Assessing longitudinal BMI trajectories provides greater power for inferring the temporal order of relationships and allows the identification of critical time points where relationships emerge or are at their strongest. Due to limited RCTs with short duration of follow-ups and inconsistent findings, our review cannot draw a causal relationship between breastfeeding and BMI trajectories. Well-designed and high-quality RCTs with longitudinal assessment of BMI are desirable. Despite that, cohort studies revealed consistent evidence supporting the beneficial association of breastfeeding and BMI trajectories. However, residual and unmeasured confounding remains an intractable issue [56, 57]. The causality between breastfeeding and BMI trajectories remains to be answered. However, our ability to test the causal relationship between breastfeeding and obesity outcomes in RCTs is hampered by ethical considerations and the cost of follow-up. Of note, a few studies assessing how breastfeeding is associated with trajectories of weight-for-height or weight z-scores are not included in the current review [[58], [59], [60], [61]]. BMI, as a universally recognized and simple measure to define and track overweight or obesity across the life course was chosen as the primary outcome of our review. However, the limitations of using BMI to monitor obesity should be noted. BMI offers no insights into body composition (e.g., body fat, lean mass). For children, monitoring overweight and obesity using BMI requires caution, and its use differs from adults. Given physical growth occurs in childhood, age and sex specific BMI z-score or percentile cut-offs are used for defining childhood overweight or obesity. Notably, cut-offs for defining childhood overweight or obesity differ by clinical assessment and research purposes. Future studies should assess trajectories of body composition to better understand the role of breastfeeding in obesity development. Given the variations in the included studies, we were unable to conduct meta-analyses to quantitatively synthesize the overall findings. However, we qualitatively summarized the findings by breastfeeding versus other feeding types, duration, breastfeeding exclusivity, and duration of follow-up. Another strength of our review is that two-thirds of included studies were rated as either high or acceptable quality, supporting the conclusion of our review. Notably, study quality did not appear to influence the significance of study findings. Studies that employed longitudinal trajectory modeling approaches were more likely to observe significant associations than those that used traditional approaches that did not account for longitudinal data. Future longitudinal studies investigating the association of breastfeeding with BMI trajectories are recommended to use longitudinal trajectory modeling. The included studies involved population of diverse geographical, socioeconomic, and ethnic backgrounds, which enhances the generalizability of our review findings.

## Conclusion

Our systematic review critically evaluated the longitudinal evidence linking breastfeeding and BMI trajectories, providing valuable insights into the long-term impact of breastfeeding versus other feeding types and breastfeeding duration on BMI trajectories over time. Children who were breastfed showed lower BMI trajectories in childhood than children who were formula-fed or mixed-fed. A longer duration of exclusive or any breastfeeding was also associated with lower BMI trajectories from childhood to early adulthood. The long-term impact of breastfeeding on development of obesity or body composition into later adulthood, underlying mechanisms, and the causality of the association, however, remains to be determined. Nevertheless, apart from the impact of breastfeeding on obesity, breastfeeding has many other health and economic benefits [4]. Our review contributes further robust longitudinal evidence from cohort studies to support infant feeding guidelines, public health initiatives, and interventions to promote and support longer duration of exclusive or any breastfeeding. Future research should be undertaken to explore potential strategies to promote breastfeeding rates and continuation at individual, community, and policy levels.

### Author contributions

The authors’ contributions were as follows—M.Z. and KJC conceived the study; M.Z. and S.G. conducted the literature search; NJDS, S.G., and M.Z. performed study screening, data extraction, and study quality assessment; M.Z. wrote the manuscript; L.A. contributed to writing of the discussion. R.L., EAS, C.G., P.B., QQH, and KJC contributed to interpretation of the study findings. All authors have read and approved the final manuscript.

### Conflict of interest

No conflict of interest to declare.

### Funding

M.Z. is supported by an Australian National Health Medical Research Council Early Career Fellowship; P.B. is supported by an Australian Research Council Future fellowship.

### Data availability

Data described in the manuscript will be made available upon request to the corresponding author.

## Appendix A Supplementary data

Multimedia component1 Multimedia component1
