# Supplementary material for: Breastfeeding and the Longitudinal Changes of Body Mass Index in Childhood and Adulthood: A Systematic Review
Source: Adv Nutr. 2023 Nov 16;15(1):100152. doi: 10.1016/j.advnut.2023.100152 (PMC10714232; doi:10.1016/j.advnut.2023.100152)
Supplement: Multimedia component1 [file mmc1.docx]

| Reference | Clearly  focused  question | Randomization  of subjects | Adequate concealment to treatment allocation | Blinding of treatment allocation to subjects and researchers | Treatment and control similar at baseline | Only difference between groups is treatment under investigation | Outcomes measure  reliable | Dropout rate of treatment arm before study completion (%) | Intent-to-treat analysis | Results comparable across study sites | Sample size justification or power description | Funding declared | Quality rating |
| --- | --- | --- | --- | --- | --- | --- | --- | --- | --- | --- | --- | --- | --- |
| Koletzko 2009 | Yes | Yes | Yes | Yes | Yes | Yes | Yes | 41% | Yes | NC | Yes | Yes | High |
| Kramer 2018 | Yes | NC | NC | NC | Yes | Yes | NC | NC | Yes | NA | Yes | Yes | Acceptable |
| Sepulveda-Valbu 2021 | Yes | NC | NC | Yes | No | Yes | Yes | 24% | No | NA | Yes | Yes | Low |

Supplementary table 1. Quality assessment for randomised controlled trials assessing breastfeeding and body mass index (BMI) trajectories

NC: not clear, NA: not applicable, Study quality assessed by Scottish Intercollegiate Guidelines Network 50 (SIGN 50) methodology checklist for randomised controlled trials.

Supplementary table 2. Quality assessment for cohort studies assessing breastfeeding and body mass index (BMI) trajectories.

| Reference | Clearly  focused  question | Subjects recruited from similar populations | Participation rate (>50%) | Drop-out rate (<20%) | Comparison  made  between  participants  and dropouts | Clearly  defined  outcome | Assessment  of outcome  blinded to  exposure | Assessment  of exposure  reliable | Validity of  outcome  measure | Repeated  exposure  measure | Adequate  adjustment  for  confounding | Confidence  interval  provided | Sample size or power | Declare of funding | Quality rating |
| --- | --- | --- | --- | --- | --- | --- | --- | --- | --- | --- | --- | --- | --- | --- | --- |
| Buyken, 2008 | Yes | Yes | NC | NC | No | Yes | Yes | Yes | Yes | Yes | Yes | Yes | No | Yes | Acceptable |
| Rhezak, 2009 | Yes | No | NC | NC | No | Yes | Yes | No | No | No | No | No | No | Yes | Low |
| Garden, 2012 | Yes | Yes | NC | NC | Yes | Yes | Yes | Yes | Yes | Yes | No | No | Yes | Yes | Acceptable |
| Jwa, 2014 | Yes | Yes | Yes | Yes | No | Yes | Yes | No | No | No | No | Yes | Yes | Yes | Low |
| Oddy, 2014 | Yes | Yes | Yes | NC | No | Yes | Yes | Yes | Yes | Yes | Yes | Yes | No | Yes | High |
| Jensen, 2014 | Yes | Yes | NC | NC | No | Yes | Yes | No | Yes | No | No | Yes | No | Yes | Low |
| Bell, 2017 | Yes | Yes | NC | NC | Yes | Yes | Yes | Yes | Yes | Yes | No | No | No | Yes | Acceptable |
| Horodynski, 2017 | No | Yes | NC | NC | No | Yes | Yes | Yes | No | Yes | No | Yes | Yes | Yes | Acceptable |
| Cheng, 2017 | Yes | Yes | Yes | NC | Yes | Yes | Yes | Yes | Yes | Yes | Yes | Yes | Yes | Yes | High |
| Rhezak, 2017 | Yes | No | NC | NC | No | Yes | Yes | Yes | Yes | Yes | Yes | Yes | No | No | Acceptable |
| Eny, 2018 | Yes | Yes | NC | NC | No | Yes | Yes | Yes | Yes | Yes | No | Yes | Yes | Yes | Acceptable |
| Huang, 2018 | Yes | Yes | Yes | NC | No | Yes | Yes | No | No | No | No | Yes | Yes | Yes | Low |
| Liu, 2018 | Yes | Yes | Yes | NC | No | Yes | Yes | Yes | No | Yes | Yes | Yes | No | No | Acceptable |
| Iguacel, 2019 | Yes | Yes | Yes | No | No | Yes | Yes | No | Yes | No | No | Yes | Yes | Yes | Low |
| Sherwood, 2019 | Yes | Yes | NC | No | No | Yes | Yes | No | Yes | No | No | No | No | Yes | Low |
| Tian, 2019 | Yes | Yes | Yes | Yes | No | Yes | NC | Yes | Yes | Yes | Yes | No | No | Yes | Acceptable |
| Zheng, 2020 | Yes | Yes | NC | NC | Yes | Yes | Yes | Yes | Yes | Yes | No | Yes | Yes | Yes | Acceptable |
| Wu, 2020 | Yes | Yes | Yes | NC | No | Yes | Yes | Yes | Yes | Yes | Yes | Yes | Yes | Yes | High |
| Florres-Barrantes, 2020 | Yes | Yes | Yes | NC | Yes | Yes | Yes | Yes | Yes | Yes | Yes | Yes | Yes | Yes | High |
| Wang, 2020 | Yes | Yes | No | NC | NC | Yes | NC | Yes | Yes | Yes | Yes | Yes | No | Yes | Acceptable |
| Zheng 2021 | Yes | Yes | NC | NC | No | Yes | Yes | Yes | Yes | Yes | Yes | Yes | No | No | Acceptable |
| Maskarinec, 2021 | Yes | Yes | Yes | NO | No | Yes | Yes | No | No | No | Yes | Yes | No | Yes | Low |
| Chen, 2022 | Yes | Yes | NA | NO | No | Yes | Yes | No | Yes | No | Yes | Yes | No | Yes | Acceptable |
| Longmore, 2022 | Yes | Yes | NC | NC | No | Yes | Yes | No | No | No | No | No | No | Yes | Low |

NC: not clear, Study quality assessed by Scottish Intercollegiate Guidelines Network 50 (SIGN 50) methodology checklist for cohort studies.
